# Role of AmpG in the resistance to β-lactam agents, including cephalosporins and carbapenems: candidate for a novel antimicrobial target

**DOI:** 10.1186/s12941-021-00446-7

**Published:** 2021-06-16

**Authors:** Roshan D’Souza, Le Phuong Nguyen, Naina A. Pinto, Hyunsook Lee, Thao Nguyen Vu, Hoyoung Kim, Hyun Soo Cho, Dongeun Yong

**Affiliations:** 1 Department of Laboratory Medicine and Research Institute of Bacterial Resistance, Yonsei University College of Medicine, 50-1 Yonsei-ro, Seodaemun-gu, Seoul, 03722 Korea; 2 J. Craig Venter Institute, Rockville, MD USA; 3 Brain Korea 21+ Project for Medical Science, Yonsei University, Seoul, Korea; 4 Department of Systems Biology, Yonsei University, Seoul, Korea

**Keywords:** AmpG, Carbapenem resistance, CRISPR–Cas9

## Abstract

**Background:**

A complex cascade of genes, enzymes, and transcription factors regulates AmpC β-lactamase overexpression. We investigated the network of AmpC β-lactamase overexpression in *Klebsiella aerogenes* and identified the role of AmpG in resistance to β-lactam agents, including cephalosporins and carbapenems.

**Methods:**

A transposon mutant library was created for carbapenem-resistant *K. aerogenes* YMC2008-M09-943034 (KE-Y1) to screen for candidates with increased susceptibility to carbapenems, which identified the susceptible mutant derivatives KE-Y3 and KE-Y6. All the strains were subjected to highly contiguous de novo assemblies using PacBio sequencing to investigate the loss of resistance due to transposon insertion. Complementation and knock-out experiments using lambda Red-mediated homologous recombinase and CRISPR–Cas9 were performed to confirm the role of gene of interest.

**Results:**

In-depth analysis of KE-Y3 and KE-Y6 revealed the insertion of a transposon at six positions in each strain, at which truncation of the AmpG permease gene was common in both. The disruption of the AmpG permease leads to carbapenem susceptibility, which was further confirmed by complementation. We generated an AmpG permease gene knockout using lambda Red-mediated recombineering in *K. aerogenes* KE-Y1 and a CRISPR–Cas9-mediated gene knockout in multidrug-resistant *Klebsiella pneumoniae*-YMC/2013/D to confer carbapenem susceptibility.

**Conclusions:**

These findings suggest that inhibition of the AmpG is a potential strategy to increase the efficacy of β-lactam agents against *Klebsiella aerogenes*.

**Supplementary Information:**

The online version contains supplementary material available at 10.1186/s12941-021-00446-7.

## Background

Gram-negative bacteria are a major threat to hospitalized patients and are associated with high mortality rates [1–4]. Hospital-acquired infections such as pneumonia, bloodstream infections, urinary tract infections, wound or surgical site infections, and meningitis are of particular concern to the clinicians. In the presence of antibiotic selective pressure, bacteria are capable of acquiring or up-regulating genes that code for antibiotic drug resistance, eventually leading to the emergence and global dissemination of these pathogens [5]. Also, Gram-negative bacteria can have redundant resistance mechanisms, either using single mechanisms against multiple antibiotics or multiple mechanisms against a single antibiotic. The antimicrobial- resistance crisis has escalated for several reasons: the high cost associated with finding novel antibiotic targets and antibiotic discovery, the length of time needed to design the drug and evaluate the efficacy, and the increased frequency of emerging bacterial resistance.

*Klebsiella aerogenes* (previously known as *Enterobacter aerogenes*) is a clinically significant bacterium in the family Enterobacteriaceae. *K. aerogenes* are part of the normal microbiota of the gastrointestinal tract in 40 to 80% of the population [6]. Though not a primary human pathogen, it has been implicated in a variety of healthcare-associated conditions, such as systemic bacteremia and urinary and lower respiratory tract infections, which are intrinsically resistant to ampicillin and narrow-spectrum cephalosporins [7, 8]. *K. aerogenes* possesses a chromosomal *ampC* β-lactamase gene that can be induced by antibiotic stress with various β-lactams. Mutations in the AmpC β-lactamase expression pathway can lead to resistance against extended- and broad-spectrum cephalosporins [9, 10].

Carbapenems, one class of β-lactam antibiotics, have historically been successful for treating cephalosporin-resistant *K. aerogenes* infections [11]. Alarmingly, *K. aerogenes* have recently emerged worldwide as carbapenem-resistant because of the high frequency of mutations in its *ampR* and *ampD* genes [12, 13]. In this study, we examined the mechanism of carbapenem resistance in *K. aerogenes* using transposon mutagenesis. In addition, we incorporated the CRISPR–Cas9-mediated gene knockout system to delete *ampG* permease and understand its role in antimicrobial resistance.

## Methods

### Bacterial strains, plasmids, antibiotics and oligonucleotides

*K. aerogenes* YMC2008-M09-943034 (KE-Y1) was collected from a tertiary-care hospital in Korea in 2008, and non-carbapenemase-producing carbapenem-resistant *Klebsiella pneumoniae* (YMC/2013/D) was collected from a different tertiary-care hospital in Korea in 2013. Bacterial identification was performed using the VITEK 32 GN system (BioMérieux, Marcy l’Etoile, France) and was confirmed using the direct colony method with MALDI-TOF MS (Bruker Daltonics, Bremen, Germany). The MICs of piperacillin, piperacillin-tazobactam, ampicillin, ampicillin-sulbactam, ceftazidime, ceftazidime-clavulanate, cefepime, imipenem, meropenem, ciprofloxacin, and aztreonam were determined by agar-dilution methods and E-test and interpreted according to the Clinical and Laboratory Standards Institute guidelines (2018). Lambda Red donor plasmid pKD46 was obtained from the *E. coli* Genetic Stock Center at Yale University (New Haven, CT, USA). Plasmid ZpUC-19 was a gift from Dr. Yo Suzuki at the J. Craig Venter Institute, La Jolla, CA, USA. Plasmids pCRISPR (Addgene plasmid # 42875) and pCas9 (Addgene plasmid #42876) were gifts from Luciano Marraffini. Plasmid pKDsgRNA-ack (Addgene plasmid #62654) was a gift from Kristala Prather. Recombinant strains were selected with spectinomycin, chloramphenicol, or kanamycin at a concentration of 50, 34, or 30 mg/l, respectively. Antibiotic concentrations were reduced by half for selecting strains harboring two or more plasmids. Oligonucleotides were synthesized by Macrogen, Inc., Korea.

### Transposon mutagenesis and complementation

Conjugation was performed by cross-streaking *E. coli* SM10 λpir and KE-Y1 on Mueller–Hinton plates. Transconjugants were selected on media containing gentamicin (2 mg/l) and ciprofloxacin (0.1 mg/l). The colonies were further replica-plated on Mueller–Hinton plates containing meropenem to obtain carbapenem-susceptible mutants. The *in-silico-*determined (see below) novel target functional gene, *ampG,* was amplified using primers AmpG-F and AmpG-R (Additional file 1: Table S3), cloned into EcoRI- and XbaI-digested pAD123 vector, and transformed into chemically competent transposon-mediated mutants (carbapenem-susceptible *K. aerogenes* KE-Y3 and *K. aerogenes* KE-Y6) as described previously [14, 15].

### DNA isolation, WGS, and analysis

The DNA of the wild-type and mutant strains were extracted using the Wizard genomic purification kit (Promega, WI, USA) with modifications to the manufacturer's protocol by adding 5 μl RNase solution during cell lysis and incubating the supernatant with DNA at − 20 °C for 1 h after addition of isopropanol. The WGS data were obtained by using the PacBio RS II sequencing system (Pacific Biosciences, Inc., Menlo Park, CA, USA) in a commercial laboratory (DNALink, Korea). To confirm the integrity of PacBio sequencing, all strains were sequenced on a 318 chip using the Ion Torrent PGM system and Ion Sequencing 200 kit (Life Technologies, Carlsbad, CA, USA). Annotations were performed using the RAST annotation pipeline with manual scrutiny [16]. All genomic analyses were performed using Geneious Pro 8.0 [17] (http://www.geneious.com).

### Amino acid alignment and AmpG structure modeling

The amino acid sequences of AmpG protein from *K. aerogenes* KE-Y1 (CP045870), *Klebsiella pneumoniae* ATCC 13883 (KN046818*)*, *Escherichia coli* O157:H7 (CP034384) and *Pseudomonas aeruginosa* PAO1 (CP053028) was aligned using Vector NTI align X. The structure model of AmpG was further established using I-TASSER according to homology modeling method [18]. The TOP 5 models predicted by I-TASSER and each model C-score are − 1.43, − 2.07, − 2.21, − 3.10, − 3.51 respectively. C-score is typically in the range of [− 5, 2], where a C-score of a higher value signifies a model with a higher confidence and vice-versa.

### CRISPR–Cas9 mediated gene knockout

*K. pneumoniae-*YMC/2013/D (carbapenemase-non-producing, carbapenem-resistant), was included for CRISPR–Cas9 mediated AmpG permease gene knockout studies. CRISPR/Cas9 system with lambda Red recombineering was used to knockout the chromosomal *ampG* permease gene as described previously [14]. First, pCas9 was transformed into electrocompetent YMC/2013/D, and clones were selected on chloramphenicol-LB agar plates. pKD46 was digested with XmnI to replace the ampicillin-resistance gene *bla*_amp_ with the spectinomycin resistance gene *bla*_spec_ to generate pKD46-spec. The spectinomycin gene was amplified from pKDsgRNA-ack using primers Spec-XmnI-F and Spec-XmnI-R. pCas9 containing YMC/2013/D was made electrocompetent again, and pKD46-spec-transformed cells were selected on spectinomycin LB plates. Plasmid pCRISPR::ampG was constructed by annealing oligonucleotides (ampG.cRNA.S and ampG.cRNA.AS) and ligating the product to BsaI-digested pCRISPR as described in the Marraffini pCRISPR protocol [19]. A 60-nt ssDNA oligonucleotide (ampG::STOP.lead or ampG:STOP.lag) encoding two consecutive stop codons in the *ampG* open reading frames and pCRISPR::ampG were mixed with 40 µl electrocompetent YMC/2013/D (pKD46-spec, pCas9). Transformants were selected using kanamycin and chloramphenicol.

### Allelic replacement mutagenesis using lambda Red recombineering

The *ampG* deletion mutant strain was constructed by gene replacement via double crossover recombination as described previously with a few modifications [20]. Briefly, three different fragments (including the upstream and downstream fragments of *ampG*) and the zeocin resistance cassette fragment were amplified using three different primer pairs i.e. Up_ampG_F/R, Zeo_F/R and Down_ampG_F/R (Table S3). The reverse primer of the upstream fragment and forward primer of the downstream fragment of *ampG* at their 5' ends included a 15–20-nt extension homologous to the primers used to amplify the zeocin marker gene. Nested overlap-extension PCR was performed using an equal concentration of the three fragments to generate a linear DNA template containing the zeocin marker gene flanked by both the upstream and downstream homologous regions. Plasmid pKD-sgRNA-ack was transformed into electrocompetent KE-Y1, and clones were screened on LB plates containing spectinomycin. Strain KE-Y1 containing pKD-sgRNA was grown in LB broth until reaching OD_600_ = 0.2, and lambda Red recombinase was expressed by adding 0.2% arabinose and with an additional 1.5-h incubation at 30 °C. Cells were harvested and made electrocompetent, and the linear DNA was electroporated. The sample was plated on low-salt LB agar plates containing 50 μg/ml zeocin and incubated at 37 °C. The *ampG* gene knockout was confirmed using accuPower PCR Premix (Bioneer, Daejeon, Korea) with different primer sets including Up_ampG_F & Down_ampG_R; Up_ampG_F & ZeoR; ZeoF & Down_ampG_R; ZeoF & ZeoR. The reaction mixture was prepared according to manufacturer instructions with 0.2 μM primer concentration..

## Results

### β-lactam susceptibility induction by mutagenesis

*K. aerogenes* KE-Y1 was mated with *E. coli* SM10 λpir containing the transposon donor vector pBTK30 encoding the aacC1 gentamicin 3'-acetyltransferase for clone selection. More than 100,000 colonies were screened to yield two transconjugants of *K. aerogenes* KE-Y1 susceptible to carbapenem, *K. aerogenes* KE-Y3 and *K. aerogenes* KE-Y6. The minimum inhibitory concentrations (MIC) of these strains are shown in Table 1. Transposon insertion led to a 16-fold decrease in ertapenem MIC (from 8 to 0.5 μg/ml) and a 32-fold decrease in the meropenem MIC (from 8 to 0.25 μg/ml). Both mutant strains were also susceptible and showed decreased MICs to piperacillin-tazobactam (32-fold), cefotaxime (64-fold), ceftazidime (64-fold), and aztreonam (64-fold).

**Table 1:** MICs of wild-type *K. aerogenes* KE-Y1 and its transposon insertion mutants (KE-Y3 and KE-Y6), its complemented derivatives, and the *ampG* knockout mutant (KE-Y1^ΔampG^)

Antibiotics	KE-Y1 Wild-type	KE-Y3 Transposon mutant	KE-Y3 + pADY123::ampG	KE-Y6 Transposon mutant	KEY6 + pADY123::ampG	KE- Y1^ΔampG^
Ampicillin	≥ 32(R)	≥ 32(R)	≥ 32(R)	≤ 2*(R*)	≥ 32(R)	≤ 4(S)
SAM	≥ 32(R)	≤ 2(R*)	≥ 32(R)	≤ 2(R*)	≥ 32(R)	≤ 2(S)
TZP	≥ 128(R)	≤ 4(S)	≥ 128(R)	≤ 4(S)	≥ 128(R)	≤ 4(S)
Cefazolin	≥ 64(R)	32(R)	≥ 64(R)	16 (R*)	≥ 64(R)	32(R)
Cefoxitin	≥ 64(R)	≥ 64(R)	≥ 64(R)	≥ 64(R)	≥ 64(R)	≥ 64(R)
Cefotaxime	≥ 64(R)	≤ 1(S)	≥ 64(R)	≤ 1(S)	≥ 64(R)	≤ 1(S)
Ceftazidime	≥ 64(R)	≤ 1(S)	32(R)	≤ 1(S)	≥ 64(R)	≤ 1(S)
Cefepime	≤ 1(S)	≤ 1(S)	≤ 1(S)	≤ 1(S)	2(S)	≤ 1(S)
Aztreonam	≥ 64(R)	≤ 1(S)	32(R)	≤ 1(S)	≥ 64(R)	≤ 1(S)
Ertapenem	≥ 8(R)	≤ 0.5(S)	≥ 8(R)	≤ 0.5(S)	≥ 8(R)	≤ 0.5(S)
Meropenem	8(R)	≤ 0.25(S)	2(I)	≤ 0.25(S)	4(R)	≤ 0.25(S)
Amikacin	≤ 2(S)	≤ 2(S)	≤ 2(S)	≤ 2(S)	≤ 2(S)	≤ 2(S)
Gentamicin	≤ 1(S)	≥ 16(R)	≥ 16(R)	≥ 16(R)	≥ 16(R)	≤ 1(S)
Levofloxacin	≤ 0.12(S)	≤ 0.12(S)	≤ 0.12(S)	≤ 0.12(S)	≤ 0.12(S)	≤ 0.12(S)
Tigecycline	1(S)	≤ 0.5(S)	≤ 0.5(S)	≤ 0.5(S)	≤ 0.5(S)	≤ 0.5(S)
TMP/SMX	≤ 20(S)	≤ 20(S)	≤ 20(S)	≤ 20(S)	≤ 20(S)	≤ 20(S)

*: AES modified (Advanced Expert system)

### Sequencing and comparative analysis

**Fig. 1:**
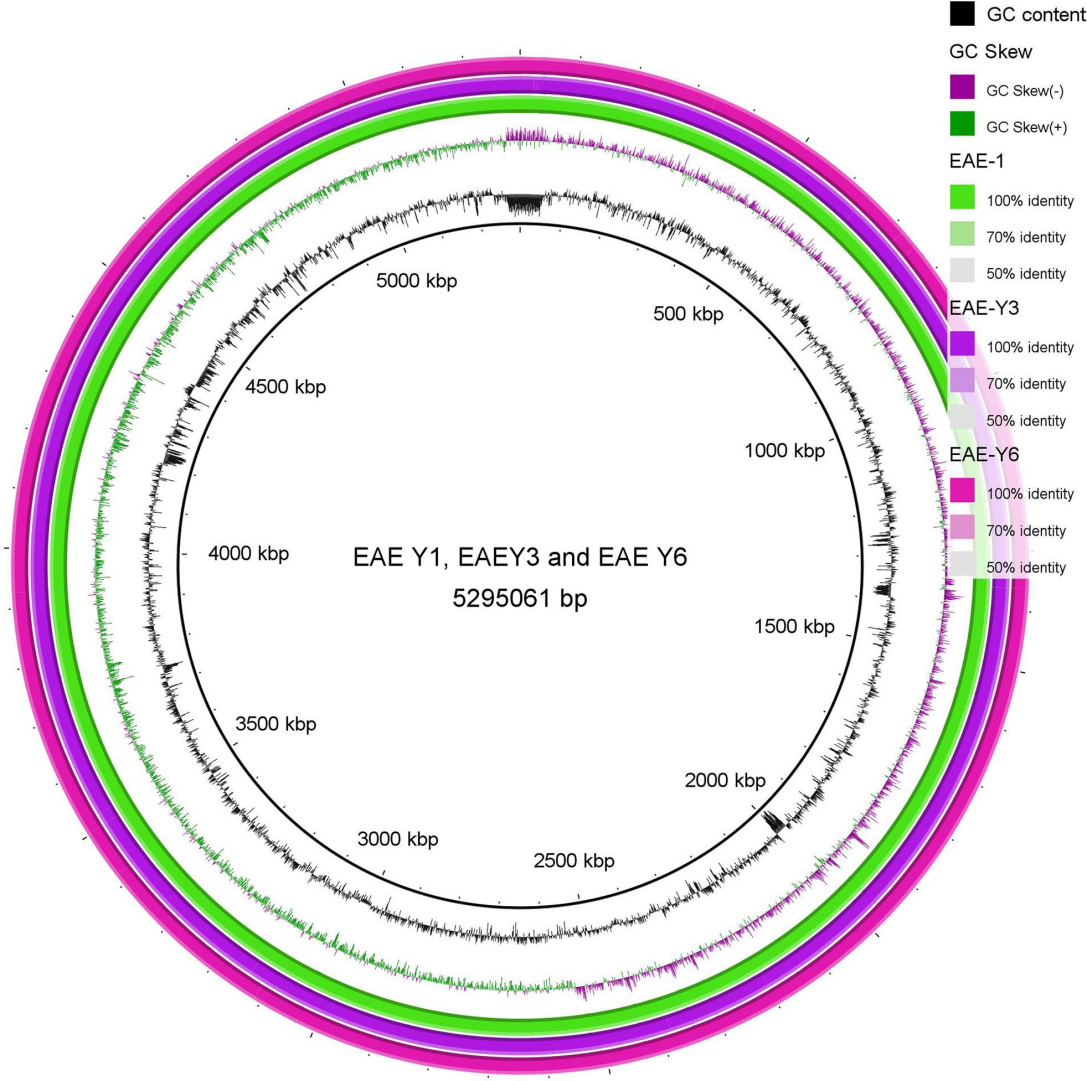
Circular view of whole-genome alignment of the chromosome of KE-Y1, KE-Y3, and KE-Y6. The first, second and third ring (outermost to inner) represent the BLAST comparisons of KE-Y1, KE-Y3, and KE-Y6 against the wildtype KE-Y1, respectively. The fourth and fifth layers indicate the GC (guanine-cytosine) skew (purple-green) and the GC content (black), respectively. The GC skew indicate the deviation from the average GC content of the three whole-genome sequences. The positions of the genome are marked in the innermost layer. This image was created using BLAST Ring Image Generator

**Fig. 2:**
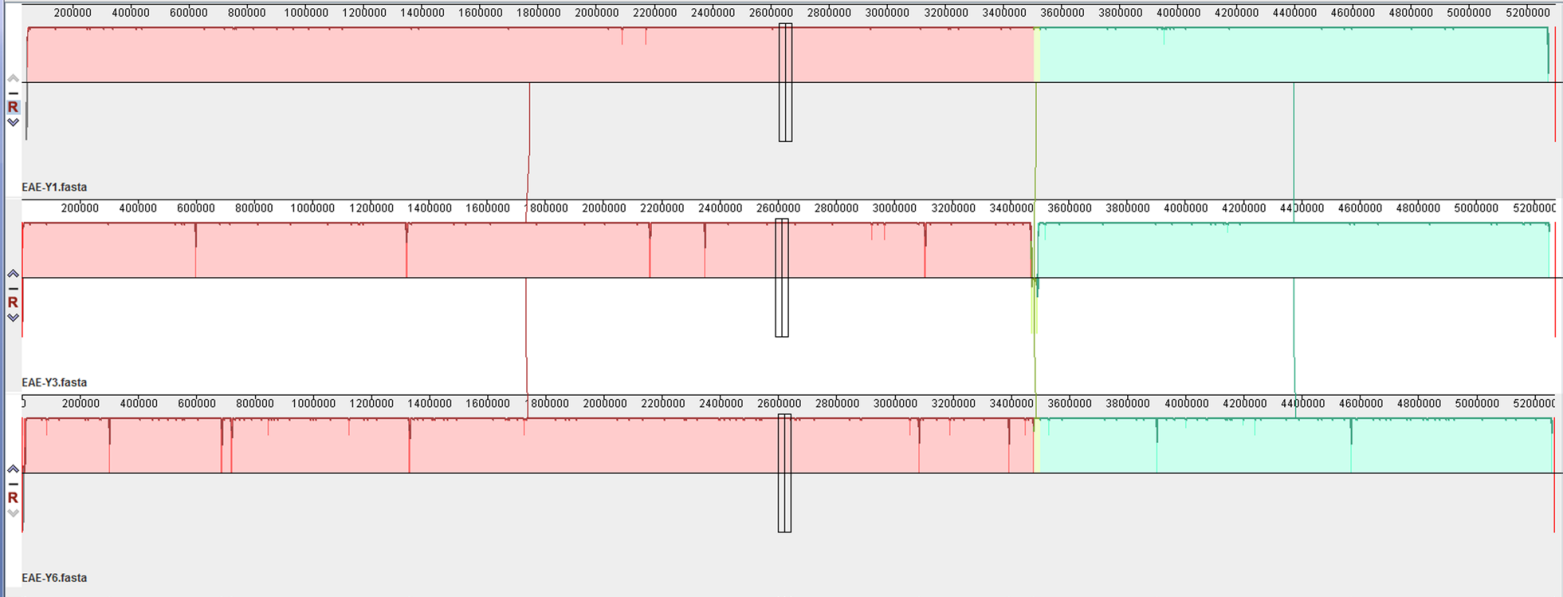
Mauve alignment of KE-Y1 (top), KE-Y3 (middle) and KE-Y6 (bottom) strains. As determined by progressive Mauve alignment, homology (colored blocks) was noted among the three strains. In the KE-Y6 strain, one locally collinear block (~ 36 Mb) was reversed; however, there was no loss of function for any genes

PacBio sequencing yielded circular genomes with 5,296,061, 5,272,156, and 5,266,224 base pairs with 54.8%, 54.9%, and 54.9% GC content for KE-Y1, KE-Y3, and KE-Y6, respectively (Fig. 1). Average Nucleotide Identity (ANI) obtained with MUMmer and BLAST indicated more than 99.8% similarity among all three strains (Additional file 1: Table S1). Whole-genome alignment using Mauve [21] indicated the homology between the three strains with one locally collinear block reversed (Fig. 2).

### Amino acid alignment and AmpG structure modeling

**Fig. 3:**
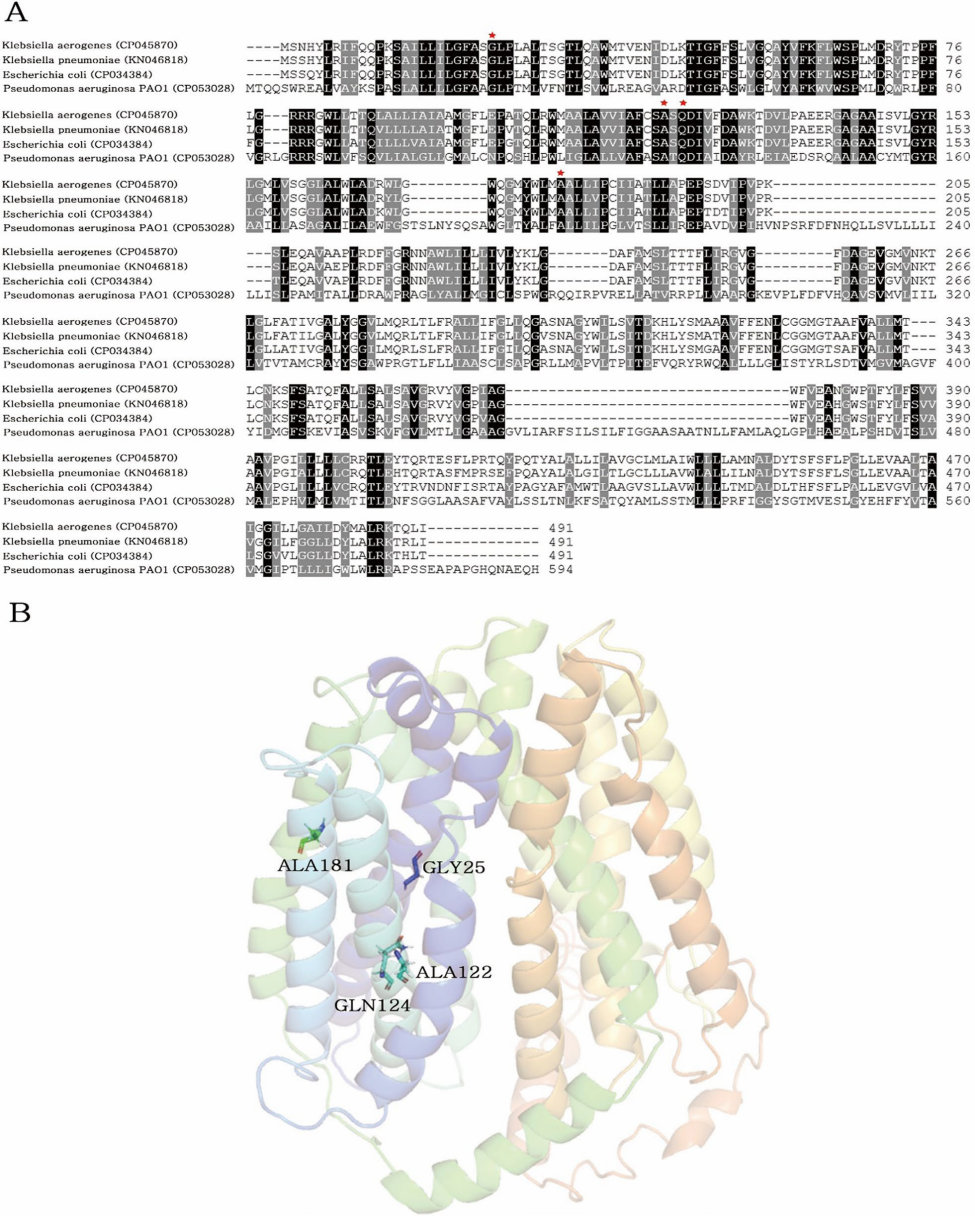
Amino acid sequence alignment and AmpG KE-Y1 structure modeling.** A** The Amino acid sequence alignment results of AmpG in 4 species; *Klebsiella aerogenes* (CP045870), *Klebsiella pneumoniae* (KN046818), *Escherichia coli* (CP034384), *Pseudomonas aeruginosa* (CP053028). Red stars are conserved activation motif residues.** B** The picture shows the homology modeling structure of AmpG *K.aerogenes* KE-Y1. The labeled residues are conserved activation motif residues

The amino acid sequence comparison of AmpG KE-Y1 with *K. pneumoniae*, *E. coli* and *P. aeruginosa* showed similarity of 96.33%, 93.08% and 41.58%, respectively. Also, all known activation motif residues (G25, A122, Q124, A181) were conserved (Fig. 3). Based on the homology modeling structure of AmpG KE-Y1, the activation motif residues are located inside the transmembrane. Therefore, it is expected to be similar to the previously known AmpG modeling structure [22].

### Open reading frames deactivated by transposon insertions

**Fig. 4:**
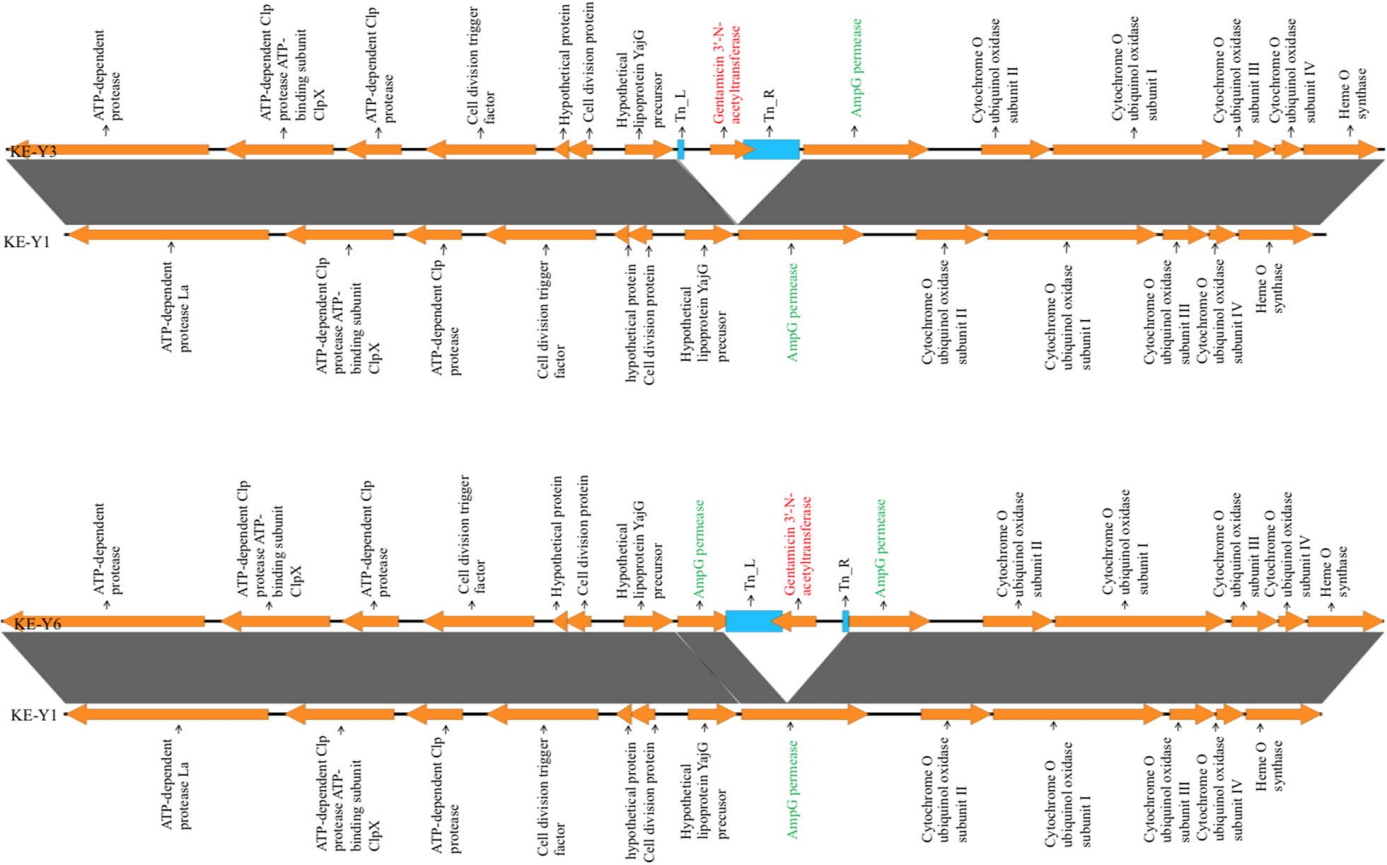
Linear comparison of multiple genomic loci around the AmpG permease between the KE-Y3 vs KE-Y1 (top) and KE-Y6 vs KE-Y1 (bottom). Both images show the transposon insertions in the KE-Y3 and KE-Y6 mutant derivatives. In KE-Y6, *AmpG* has been truncated in the middle due to the insertion of transposon. However, in strain KE-Y3, transposon insertion is immediately upstream of *AmpG,* resulting in the loss of the promoter required for its expression

**Fig. 5:**
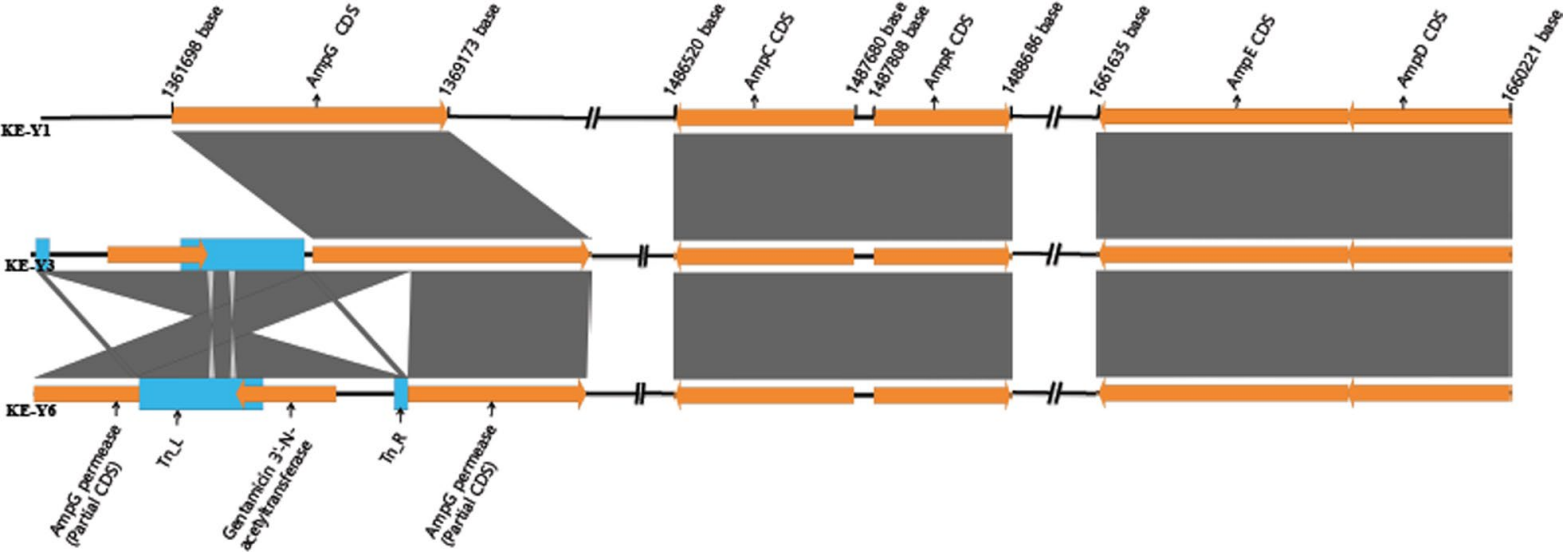
Diagram indicating the genes responsible for peptidoglycan recycling. This illustrates the position of the AmpG-AmpC-AmpR-AmpD gene network in the whole genome of KE-Y1, KE-Y3, and KE-Y6. The AmpG permease coding sequence (CDS) has been interrupted by the transposon (blue) in KE-Y3 and KE-Y6

Whole-genome sequencing (WGS) analysis of KE-Y1, KE-Y3, and KE-Y6 revealed six insertions of gentamicin-encoded transposons. The transposon-mediated inactivated genes are listed in Additional file 1: Table S2. AmpG permease gene insertions were seen in both carbapenem-susceptible transposon insertion strains KE-Y3 and KE-Y6. In strain KE-Y3, the AmpG permease gene was truncated in the middle by the transposon insertion. However, in strain KE-Y6, the transposon inserted immediately upstream of the gene, leading to the loss of the promoter required for AmpG permease gene expression (Fig. 4). The genome position of the genes responsible for induced β-lactamase expression is illustrated in Fig. 5, along with its comparison to that of the wild-type and its transposon mutant derivatives. AmpC-AmpR and AmpD-AmpE are closely linked with each other. The localization of the AmpG, AmpC-AmpR, and AmpD-AmpE genes are similar and identical in all the three strains.

### AmpG permease and carbapenem resistance

Complementation of susceptible strains KE-Y3 and KE-Y6 with plasmid pAD123::AmpG reverted the strain's resistance phenotype. We observed a 16-fold (0.5 to 8 μg/ml) increase in ertapenem resistance for both KE-Y3 and KE-Y6, and meropenem resistance was increased eightfold (0.25 to 2 μg/ml) and 16-fold (0.25 to 4 μg/ml) for both strains, respectively. In addition, the complemented strains were resistant to piperacillin-tazobactam (≥ 128 μg/ml), cefotaxime (≥ 128 μg/ml), ceftazidime (≥ 32 μg/ml), and aztreonam (≥ 32 μg/ml). *AmpG* gene knockout from the wild-type KE-1 using allelic replacement mutagenesis induced susceptibility to most of the antibiotics tested, and the MICs were similar to the transposon mutants KE-Y3 and KE-Y6.

### CRISPR–Cas9 mediated AmpG permease knockout

The clinical isolate *K. pneumoniae-*YMC/2013/D was included for CRISPR–Cas9-mediated gene knockout studies to evaluate the role of AmpG permease in another Gram-negative multidrug resistant pathogen. The CRISPR/Cas9 knockout system, coupled with lambda Red recombineering, has been used to overcome cell death caused by double-stranded DNA breaks in Enterobacteriaceae. An AmpG knockout in *K. pneumoniae-*YMC/2013/D induced susceptibility to carbapenems. MIC data indicated a ≥ fourfold (≥ 0.32 to 8 μg/ml) and twofold (8 to 4 μg/ml) decrease in resistance to imipenem and meropenem, respectively, compared to the wild-type isolate.

## Discussion

Our results demonstrate that carbapenemases do not mediate carbapenem resistance in *K. aerogenes*. Thus, control and management of carbapenem resistance should not be focused solely on the use of carbapenemases. Carbapenem resistance can also be due to overexpression of AmpC, efflux pumps, and porin loss (or a combination of these). To the best of our knowledge, this is the first report of *K. aerogenes* and the role of AmpG permease on carbapenem resistance following induction of *ampC* during antibiotic-induced stress. This stress may lead to many other mutations in the bacterial genome that cause resistance to a wide variety of non-carbapenem antibiotics such as cefotaxime, ceftazidime and cefepime [10].

AmpC is a chromosomally encoded group I, class C cephalosporinase produced by *K. aerogenes* at basal levels. The presence of β-lactams, such as cefoxitin and imipenem, highly induce AmpC expression [23], which involves a complex network of regulatory genes closely linked with peptidoglycan recycling [24]. During antibiotic treatment, the balance of peptidoglycan synthesis is compromised, releasing GlcNAc-anhydro-MurNAc-oligopeptides into the periplasm. Resulting murapeptides are transported into the cytosol by an AmpG transporter encoded by *ampG* [25]. However, AmpG has no influence on *ampC* induction, nor does it show a gene dosage effect [26]. Upon entry into the cytosol, the GlcNAc sugar residue is removed by β-N-acetylglucosaminidase (NagZ) to generate 1,6-anhydromuropeptide, which is processed by N-acetyl-anhydromuramyl-L-alanine amidase (AmpD) during the non-induced state [27, 28]. However, growth in the presence of β-lactams leads to increased breakdown of peptidoglycan or mutations in the *ampD* gene and may eventually lead to an increased intracellular concentration of murapeptides [29]. Another gene, *ampE*, located near *ampD*; AmpD modulates the response exerted on β-lactamase expression by AmpE [30].

In the carbapenem-resistant KE-Y1 wild-type strain, this increased concentration of intracellular murapeptides might have induced the AmpC production by interacting with the LysR-type transcriptional regulator AmpR, making the strain resistant to carbapenems [31]. The *ampR* gene is located immediately upstream of *ampC* and encodes a DNA-binding protein that activates *ampC* [32, 33].

Consistent with the above observations and data obtained in this study, the *ampG* knockout implicates this protein as a potential pharmaceutical target for controlling *ampC* hyper-expression. Studies indicate that cells lacking AmpD or AmpG lose 40% of the peptidoglycan layer per generation [31]. Both carbapenem-susceptible transposon insertion mutants KE-Y3 and KE-Y6 contained an *ampD* gene; therefore, the susceptibility to the carbapenem was due to the loss of functional *ampG*. Similar studies previously suggested the role of AmpG in the antimicrobial resistance of *P. aeruginosa* and *K. cloacae *[26, 34].

## Conclusions

To our knowledge, this is the first report illustrating the role of AmpG in carbapenem resistance in *K. aerogenes.* We used knockout studies with transposon mutagenesis in *K. aerogenes* KE-1 and the CRISPR–Cas9 gene knockout system to inactivate AmpG permease in a multidrug-resistant clinical isolate of *K. pneumoniae.* Additionally, using gene complementation, we reversed carbapenem resistance to validate our findings. Future studies should explore additional AmpG protein inhibitors as therapeutic drugs for controlling antibiotic resistance. Also, the other inactivated genes in our study due to mutagenesis should be further analyzed to detect any additional change in the phenotype. The transposon mutagenesis approach could be used to understand novel resistance mechanisms in other classes of bacteria to potentially identify other antibacterial targets.

## Supplementary Information

**Additional file 1: Table S1. **Average nucleotide analysis using BLAST and MUMmer. **Table S2.** List of genes interrupted due to transposon insertion in the mutagenized strains. **Table S3.** Oligonucleotides used in this study.

## Data Availability

The WGS data of KE-Y1, KE-Y2, and KE-Y3 generated by the PacBio RS II sequencing system are under the GenBank accession numbers CP045870, CP045869 and CP045868, respectively.
