# Supplementary material for: Role of AmpG in the resistance to β-lactam agents, including cephalosporins and carbapenems: candidate for a novel antimicrobial target
Source: Ann Clin Microbiol Antimicrob. 2021 Jun 16;20:45. doi: 10.1186/s12941-021-00446-7 (PMC8207665; doi:10.1186/s12941-021-00446-7)
Supplement: Supplementary file 1 — Additional file 1: Table S1. Average nucleotide analysis using BLAST and MUMmer. Table S2. List of genes interrupted due to transposon insertion in the mutagenized strains. Table S3. Oligonucleotides used in this study. [file 12941_2021_446_MOESM1_ESM.docx]

**Table S1.** Average nucleotide analysis using BLAST and MUMmer.

|  | Strain KE-Y1 | | Strain KE-Y3 | | Strain KE-Y6 | |
| --- | --- | --- | --- | --- | --- | --- |
|  | BLAST | MUMmer | BLAST | MUMmer | BLAST | MUMmer |
| KE -Y1 | --- | --- | 99.97 | 99.89 | 99.96 | 99.88 |
| KE -Y3 | 99.96 | 99.93 | --- | --- | 99.95 | 99.91 |
| KE -Y6 | 99.95 | 99.88 | 99.95 | 99.87 | --- | --- |

**Table S2.** List of genes interrupted due to transposon insertion in the mutagenized strains.

| KE-Y3 | KE-Y6 |
| --- | --- |
| Acyltransferase RutD | 2-dehydropantoate 2-reductase aldehyde dehydrogenase |
| AmpG permease CDS | Flagellar hook protein FlgE CDS |
| Transcriptional regulator PhnF CDS | Oligopeptide ABC transporter |
| Zinc-resistance associated protein CDS | AmpG permease CDS |
| Acetyltransferase (EC 2.3.1.) CDS | None |
| Sucrose-specific transcriptional regulator | Putative inner membrane protein YqgA CDS |
| PEP protein phosphotransferase | None |

**Table S3.** Oligonucleotides used in this study.

| **Primer/crRNA** | **Sequence (5’-3’)** |
| --- | --- |
| AmpG-F | GAATTCTAAAATAGCAGACCCGGTATC |
| AmpG-R | TCTAGATTAAATCAGCTGCGTTTTGC |
| Up_ampG_F | CGCGATGTTTGTACTCGCAG |
| Up_ampG_R | GTGATGACGGTGAAAACCTCGGCATATAGAGCAGACCCGG |
| Zeo_F | GAGGTTTTCACCGTCATCAC |
| Zeo_R | CTCATAGCTCACGCTGTAGG |
| Down_ampG_F | CCTACAGCGTGAGCTATGAGCGCCAAACCGTTAATAGCCG |
| Down_ampG_R | GGCGTTTAAAGTGTACGGGC |
| Nest_Fwd | TTACCGTCAGCATCAACG |
| Nest_Rev | ATGTTACGGAATGTGACG |
| ampG.cRNA.S | aaacGGTATTACTCCTGCTGGCGATCGCGGCGATg |
| ampG.cRNA.AS | aaacATCGCCGCGATCGCCAGCAGGAGTAATACC |
| ampG::STOP.lead | CTTGGGCGTCGTCGCGGTTGGCTGTTGACCACCCAGGTATTACTCCTGCTGGCGATC  GCGACGtaataaTTTCTTGAGCCGGTCACCCAATTACGCTGGATGGCGGCGCTGGCGGTGGTGATCGCCTTCT |
| ampG::STOP.lag | AGAAGGCGATCACCACCGCCAGCGCCGCCATCCAGCGTAATTGGGTGACCGGCTCA  AGAAAttattaCGTCGCGATCGCCAGCAGGAGTAATACCTGGGTGGTCAACAGCCAACCGCGACGACGCCCAAG |
| Spec-XmnI-F | GAANNNNTTCTTATTTGCCGACTACCT |
| Spec-XmnI-R | GAANNNNTTCATGAGGGAAGCGGTGAT |
